# The Great Need

**Published:** 1913-05-15

**Authors:** A. W. Thornton

**Affiliations:** Toronto, Ont.


### THE GREAT NEED.

BY A. W. THORNTON, D.D.S., L.D.S., TORONTO, ONT.

Delivered at the banquet given by the Dentists of Pittsburgh to the Institute of Dental Pedagogics.

Mr. Chairman: I presume that there is not a man in this room to-night, who has not at some time felt a longing, a desire, to possess the power, that by common consent, is ascribed to the world's great public speakers, the power to stir men out of themselves, to make the blood pulsate, the breath to quicken, the eye to glisten, to move men so that they see with the speaker's eye, feel as the speaker feels, and become obsessed with an overmastering desire to act in harmony with the expressed will of the speaker. I confess honestly, that as I thought of speaking to the men who are here to-night, some such longing took possession of me. But I realized as fully as any of you do, how absolutely impossible it would be for me to exercise any such influence. Such power is bestowed upon but few men, it is the divine gift, the gift of the Gods. What then must common mortals do? Must we refrain from saying anything, because we do not possess the wisdom of a Solon, the eloquence of a Burke, the homely magnetism of a Lincoln? No, the world today is not so much concerned with ornate language, beauty of diction, or the force or elegance of an epigram, as it is with the integrity of purpose, and depth and honesty of conviction of a man who presumes to speak to his fellowmen, with the idea of influencing their conduct along any line of action. This then is my only hope, indeed it is my only warrant for speaking to you at all, the fact that I am speaking to you from the depth of my heart my own honest conviction.

I need not remind the gentlemen here present of the evolution of the profession of Dentistry. From the lowliest and crudest of beginnings, from the peripetetic dentist with a few instruments, going from village to village, extracting teeth and making dentures, from the time of indentures and apprenticeship, from all of these, to the position of Dentistry at the present day, is indeed a far cry. To-day we have the man with well trained mind, fitted in college, wherein no expense is spared, that the best possible training may be given to the student preparing to enter the profession; we have the most estensive and expensive manufacturing plants, turning out instruments and appliances, almost human in their perfection. We have changed and modified and improved until it would almost seem that we have reached the limit of possibility, the point at which we might exclaim "ne plus ultra," nothing beyond. And yet I have dared to suggest, what I believe many of you feel, that there is still "a great need." Over the door of every Dental Office, in every town and city on this continent; over the door of every Dental College in the sanctum of every editor of a Dental Journal, the discerning eye may read the familiar words, "men wanted."

I know that you will not misunderstand me. I know as well as you know, that in private practice, in college, and in professional journalism, we have many grand and capable men, but their number is all too few, for the Herculean tasks that must be undertaken. If the profession of Dentistry is not to be thoroughly and absolutely commercialized, the most stupendous efforts must be put forth to combat the present day tendencies.

And you men, you who are teachers, must undertake the task.

I need not remind you of the materialistic tendencies of the age. Everybody, everywhere, is worshipping the golden calf. And the general feeling is, that this materialism, this money worship, dominates the professional man to-day, as thoroughly as it does the man in a purely commercial business. The feeling in the lay mind to-day is that the lawyer exploits his client, and advises litigation, simply that he may obtain a fee; that in the medical profession, thousands of operations are performed, and the credulity of the uninitiated traded upon, solely that the surgeon and medical man may profit, and that the dentist is advising and inserting unnecessary and expensive operations, from which patients do not receive value. Our boys are going out from our colleges to-day and the thought uppermost in their minds is "Dr. Smith made $5,000 last year, Dr. Jones made $10,000, Dr. Robinson made $20,000." Not a thought of the service rendered to the people who paid these amounts. Saddest of all is the thought, that there is not in the minds of many of our students a desire to equip themselves, so as to render the service that will command these large fees. The great thought is "get the fee," regardless of the worth of the operation. We hear so often the words "how much," "how much," "how much," that our very souls grow sick of them.

Once again let me beg of you not to misunderstand me. I know, we all know, that a dentist, in common with every other professional man, owes it to himself to look carefully after the business end of his practice. His fees should be sufficiently large, and his accounts so promptly collected, that he can live comfortably, make some provision for the proverbial rainy day, and promptly meet his obligations. But all this may be done, without making the bigness or the smallness of the fee the chief thing to be considered. I very much doubt the wisdom of Brother Tom, or Brother Dick, or Brother Harry, or Brother anybody else, sowing this country knee deep with literature, insidiously appealing to the cupidity, the selfish nature of the men of the profession, a part of our nature all too prone to run riot, without any such stimulation.

Now I trust that none of you in talking of this subject will say, "Oh, Thornton was talking through his hat, or speaking to the grand stand." We must have fees, and we should have large fees, for I know of no calling that demands a higher standard of intelligence and more constant application, a calling more exacting in its demands than the faithful conscientious practice of Dentistry.

But there are some things that come before the fees. We must have the skill, the ability, to render the service that will be worthy of large fees, and the service must be rendered, patients should receive value for their money. More than that, let me say, I believe there is a better way to obtain large fees, than many of the ways that are being followed. If the dentists of this country, could once convince the public that the advice given, the service rendered, was absolutely honest and unselfish, if men and women could come to a dentist and say "do what you think is best, I know you will do what is right," we could obtain a practice, both in volumn and in value, such as few of us have dreamed of.

Is it possible to bring about this condition? I do not know that it is, but I do know, that there must be in all our schools, and all the surroundings, of our teachers, an atmosphere that will be helpful in the production of such a professional standard and spirit.

We must teach our students to have the most profound respect for the sacred nature as well as the perfection of the human body, and that they must not ruthlessly mutilate any portion of it. They must be taught to realize too, the difficulty and delicacy of our operations, so that they may give themselves to the arduous task of proper preparation. They must learn too, that our patients come to us, very often suffering, and always ignorant of the cause, and that they are absolutely at our mercy, and that to take advantage of their suffering, their ignorance, or their credulity, is more contemptible than the petty theft of the man who robs the clothes line, and more cowardly than the act of the highwayman, who robs an express car, or sandbags a millionaire.

In the name of God, let us teach our boys not to look upon the fee, as the only thing to be sought after, regardless of the value of the operation, its approach to some degree of perfection, or the patient's ability to pay for it.

"God give us men, a time like this demands,
Strong minds, great hearts, true faith, and ready hands.
Men whom the lust of office does not kill,
Men whom the spoils of office cannot buy,
Men who possess opinions and a will,
Men who have honor, men who will not lie,
Men who can stand before a demagogue,
And scorn his treacherous flatterings without winking,
Tall men, sun crowned, who live above the fog
In public duty, and in private thinking."—*Dominion Journal.*

